# Total glycosides contribute to the anti-diarrheal effects of Qiwei Baizhu Powder *via* regulating gut microbiota and bile acids

**DOI:** 10.3389/fcimb.2022.945263

**Published:** 2022-08-22

**Authors:** Guozhen Xie, Na Deng, Tao Zheng, Xinxin Peng, Shuihan Zhang, Zhoujin Tan

**Affiliations:** ^1^ School of Pharmacy, Hunan University of Chinese Medicine, Changsha, China; ^2^ College of Traditional Chinese Medicine, Hunan University of Chinese Medicine, Changsha, China; ^3^ The First Hospital of Hunan University of Chinese Medicine, Changsha, China; ^4^ Institute of Chinese Materia Medica, Hunan Academy of Chinese Medicine, Changsha, China; ^5^ College of Medicine, Hunan University of Chinese Medicine, Changsha, China

**Keywords:** total glycosides of Qiwei Baizhu Powder, gut microbiota, bile acid metabolism, diarrhea, bioactive component

## Abstract

Qiwei Baizhu Powder (QWBZP) is a traditional Chinese medicine formula for treating diarrhea induced by various causes. It elicits an anti-diarrheal effect by regulating the gut microbiota (diversity, structure, and abundance). However, the contribution of different components in the QWBZP decoction to this effect remains unclear. In this study, we used the QWBZP decoction as a reference standard to investigate the effects of total glycosides (TGs) extracted from QWBZP decoction on the gut microbiota and bile acid metabolism in mice with antibiotic-associated diarrhea (AAD). The results of 16S rRNA gene sequencing and liquid chromatography-mass spectrometry (LC-MS) analysis showed that the effect of total glycosides of Qiwei Baizhu Powder (QWBZP-TG) on specific intestinal bacteria and bile acids was similar to that of the QWBZP decoction, but the intensity of this effect was more significant in the case of QWBZP-TG. The QWBZP decoction and QWBZP-TG promoted the proliferation of *Lactobacillus* and inhibited the growth of *Proteus*, *Clostridium*, *Eubacterium*, *Facklamia*, and *Escherichia* in AAD mice. They also increased the levels of deoxycholic acid and beta-muricholic acid and decreased those of taurocholate acid, tauro-alpha-muricholic acid, and tauro-beta-muricholic acid in AAD mice. *Lactobacillus* was the key bacterial genus responding to QWBZP-TG. Thus, this study provides novel insights into the bioactive components of QWBZP and their contribution to its effects.

## Introduction

Numerous microorganisms widely colonize the gut, and contribute to nutrition and drug metabolism, maintain intestinal barrier immunity, regulate host immunity, and resist pathogens invasion. As dysbiosis is the underlying cause of multiple diseases, maintenance of gut microbiota homeostasis is essential for maintaining health (Adak and Khan, 2019; Kc et al., 2020). Antibiotic-associated diarrhea (AAD) is defined as clinically unexplained diarrhea induced in response to antibiotic administration (Mekonnen et al., 2020). Diarrhea and dysbiosis often influence each other, i.e., dysbiosis induces diarrhea, which leads to the release of numerous intestinal bacteria, results in an imbalance in intestinal microecology, and interferes with the metabolic function of intestinal bacteria, and all these phenomena result in the further aggravation of diarrhea. Therefore, the gut microbiota is a key target for AAD treatment (Li et al., 2021).

Bile acids (BAs), one kind of endogenous signaling molecule, are produced in the liver from cholesterol and metabolized in the intestine by the gut microbiota (Wahlström et al., 2016). BAs crosstalk with the gut microbiota. For instance, the gut microbiota metabolizes primary BAs [cholic acid (CA), chenodeoxycholic acid (CDCA), ursodeoxycholic acid (UDCA), alpha-muricholic acid (alpha-MCA), and beta-muricholic acid (beta-MCA) in mice] into secondary BAs [such as deoxycholic acid (DCA), lithocholic acid (LCA), iso-BAs, and allo-BAs] by deconjugation, dehydroxylation, dehydrogenation, or epimerization (de Vos et al., 2022). BAs in turn regulate microbial composition through direct antimicrobial effects and indirect effects *via* X-activated receptor (FXR)-induced antimicrobial peptides (Ridlon et al., 2014). Importantly, the modulation of BAs on the gut microbiota is related to its structure. Unconjugated and secondary BAs are more hydrophobic than the corresponding conjugated and primary BAs, respectively. Therefore, unconjugated and secondary BAs are more likely to exert direct antimicrobial activities through membrane damage (Cai et al., 2022). Furthermore, BAs play a pivotal role in regulating glucose, lipid, and energy metabolism, as well as affect gastrointestinal motility and the secretion of water electrolytes and mucus in the intestine (Wei et al., 2020; de Vos et al., 2022). Disorders in BA metabolism are observed in individuals with irritable bowel syndrome, inflammatory bowel disease, short bowel syndrome, *Clostridium difficile* infection, AAD, and other intestinal diseases (Duboc et al., 2012; Joyce and Gahan, 2017; Wei et al., 2020; Mekonnen et al., 2020). Thus, microbiota-BAs interactions play an important role in regulating host metabolism and immunity (Wahlström et al., 2016; Joyce and Gahan, 2017; Urdaneta and Casadesús, 2017).

Qiwei Baizhu Powder (QWBZP), first recorded in Xiaoer YaoZheng ZhiJue, is composed of Atractylodis Macrocephalae Rhizoma (Baizhu), Ginseng Radix et Rhizoma (Renshen), Poria (Fuling), Puerariae Lobatae Radix (Gegen), Aucklandiae Radix (Muxiang), Pogostemonis Herba (Guanghuoxiang), and Glycyrrhizae Radix et Rhizoma (Gancao) (Qian, 2008). QWBZP is an ancient traditional Chinese medicine (TCM) formula for diarrhea treatment and is now widely used to treat gastrointestinal diseases, including AAD (Peng et al., 2014). Previous study revealed that QWBZP ameliorates AAD by regulating the intestinal microecology and exerting anti-inflammatory effects (Hui et al., 2020; Long et al., 2020). TCM-mediated disease treatment involves multi-components, multi-targets, and multi-paths. Thus, the contribution of individual whole TCM and/or formula components should be understood.

In clinical practice, QWBZP is most frequently used in the form of decoction, whose dominant components are glycosides. In a previous study, we extracted total glycosides (TGs) from QWBZP (QWBZP-TG), and using *in vitro* culture technology, confirmed that QWBZP-TG promotes the proliferation of *Bifidobacteria* and *Lactobacillus* in AAD mice. QWBZP-TG exhibits effects similar to those of QWBZP (Xie et al., 2021). Therefore, QWBZP-TG represents the bioactive component that mediates the anti-diarrheal effects of QWBZP. Considering the crosstalk between the gut microbiota and BAs, we hypothesized that the QWBZP decoction and QWBZP-TG would be able to regulate gut dysbiosis and BA metabolism in AAD mice. The similarities and differences between the QWBZP decoction and QWBZP-TG should be compared in terms of their regulatory effects on the gut microbiota and BA metabolism to clarify the contribution of TGs to QWBZP efficacy.

In this study, the QWBZP decoction was taken as a reference, and the effects of QWBZP-TG on gut microbiota and BA metabolism were investigated using 16S rRNA gene sequencing and liquid chromatography-mass spectrometry (LC-MS) technology, respectively, to further elucidate the role of the bioactive components and mechanisms by which QWBZP acts in the context of AAD therapy. This study might provide a meaningful basis for future research on “whole and part relationships” in TCM formulas and associated compounds.

## Materials and methods

### Materials and reagents

Seven Chinese herbal slices of QWBZP, namely, Atractylodis Macrocephalae Rhizoma (20201014, Hunan, China), Ginseng Radix et Rhizoma (20200610, Jilin, China), Poria (20201109, Hunan, China), Puerariae Lobatae Radix (20201020, Hunan, China), Aucklandiae Radix (20201011, Yunnan, China), Pogostemonis Herba (20200816, Guangdong, China), and Glycyrrhizae Radix et Rhizoma (20200610, Neimenggu, China), were purchased from The First Hospital of Hunan University of Chinese Medicine and certified by Qingping Pan, a professor at the School of Pharmacy, Hunan University of Chinese Medicine. Gentamicin sulfate (01Y07011A2) and cefradine (06200502) were purchased from Yichang Renfu Pharmaceutical Co. Ltd. (Yichang, China) and Jilin Wantong Pharmacy Group Co. (Jilin, China), respectively.

### Preparation of QWBZP and QWBZP-TG

All the Chinese herbal slices of QWBZP were mixed and boiled twice using a 10-fold mass of distilled water for 30 min each time. The decoction was combined after filtration and concentrated at 0.34 g/mL. The QWBZP decoction was stored at 4°C and reheated to 30°C before use. QWBZP-TG was prepared in accordance with our optimized method (Xie et al., 2021). QWBZP-TG powder was dissolved in sterile water and mixed homogeneously. The QWBZP-TG solution was stored at 4°C and was reheated to 30°C before use.

### Animals and treatments

Male Kunming mice (specific pathogen-free, five weeks old, 20 ± 2 g) were provided by Hunan Slaccas Jingda Laboratory Animal Co., Ltd. (Changsha, China) with license number SCXK (Xiang) 2019-0004. The mice were housed at the Animal Experiment Center of Hunan University of Chinese Medicine (Changsha, China). The experiments were approved by the Animal Care and Use Committee of Hunan University of Chinese Medicine (authorization number: LL2020102103). Standard food and purified water were provided ad libitum.

After three days of acclimation, 28 mice were randomly divided into two groups: a normal control group (N, 7 mice) and an AAD group (AAD, 21 mice). AAD models were established in accordance with our optimized method (Hui et al., 2020). In detail, AAD mice were administered a mixture of gentamycin sulfate and cefradine (62.5 mg/mL, 0.35 mL) twice per day for five days to establish AAD models. Normal control mice were administered 0.35 mL sterile water. After five days of modeling, the AAD mice were further randomly divided into three groups (7 mice/group): (1) restore group (R), treated with sterile water; (2) QWBZP decoction group (TW), treated with QWBZP; (3) QWBZP-TG group (TG), treated with QWBZP-TG. According to the clinical equivalent dose, the dosage of QWBZP was 9.945 g/kg (body weight) [BW]/d. Based on the results of previous study (Xie et al., 2021), the dosage of QWBZP-TG was 147.2 mg/kg (BW)/d, which is equal to the clinical dose of QWBZP multiplied by the extraction rate of QWBZP-TG. The administered volume was 0.4 mL for each mouse, twice per day for three days.

### Sample collection

After treatment, the mice were sacrificed by cervical dislocation. Under aseptic conditions, small intestine contents (from duodenum to ileum) and colonic feces were collected from each mouse using sterile tweezers. All samples were immediately placed in sterile cryopreservation tubes, frozen in liquid nitrogen, and stored at -80°C until DNA extraction and BA analysis.

### 16S rRNA gene sequence

Genomic DNA was extracted from the small intestine contents using a DNA isolation kit (Omega, USA) according to the manufacturer’s instructions. The concentration and purity of the extracted genomic DNA were measured using Nano-drop 2000 (Thermo Fisher Scientific, USA), and quality was determined by electrophoresis on 1% agarose gel. The V3-V4 hypervariable regions of the 16S rRNA genes were amplified using PCR with the following primers: 338F (5’-ACTCCTACGGGAGGCAGCAG-3’) and 806R (5’-GGACTACHVGGGTWTCTAAT-3’). Amplicon pyrosequencing was performed on the DNA samples using the Illumina MiSeq platform (Illumina, San Diego, USA).

Demultiplexed sequences from each sample were quality filtered and trimmed, de-noised, merged, and then the chimeric sequences were identified and removed using the Quantitative Insights into Microbial Ecology version 2 (QIIME2) dada2 to obtain clean sequences (Callahan et al., 2016). Clean sequences were assigned to the same operational taxonomic units (OTUs) with ≥ 97% similarity and classified against Greengenes (Greengenes Database 13_8). Alpha diversity indices, including Chao1, Faith’s phylogenetic diversity (Faith_pd), Shannon, and Simpson, were calculated using QIIME2. Beta diversity analysis was performed using principal coordinate analysis (PCoA) based on the Bray-Curtis distance. Linear discriminant analysis effect size (LEfSe) was conducted to identify microbial structure differences among groups. The data presented in the study are deposited in the NCBI repository, accession number PRJNA846967.

### BA analysis

Colonic fecal samples were thawed at room temperature before preparation. Each sample was mixed with 400 μL of methanol by vortexing for 60 s. Then, the samples were ground twice with glass beads at 55 Hz for 60 s, exposed to ultrasound for 30 min, and centrifuged at 12,000 rpm for 10 min. Following this, 300 μL supernatant and 600 μL ultrapure water were mixed by vortexing for 30 s. Then, 50 μL of supernatant and 950 μL of 30% methanol were mixed homogeneously and filtered using a 0.22 μm microporous membrane before analysis.

Chromatographic analysis was performed on a Shimadzu LC-30 (Shimadzu, Japan). Chromatography was conducted using an ACQUITY UPLC^®^ BEH C18 column (2.1 × 100 mm, 1.7 μm, Waters, United States) with a constant flow rate of 0.25 mL/min at 40°C. The mobile phase was composed of 0.01% formic acid water (A) and acetonitrile (B). Gradient elution was set as follows: 70% A for 0-9 min, 70%-64% A for 9-14 min, 64%-62% A for 14-18 min, 62%-50% A for 18-24 min, 50%-25% A for 24-32 min, 25%-10% A for 32-32.01 min, 10% A for 32.01-33 min, 10%-70% A for 33-33.01 min, 70% A for 33.01-35.5 min. The injection volume was 5 μL.

Mass spectrometry (MS) with an electrospray ionization (ESI) source operating in a negative ion mode was conducted on an AB 6500Plus mass spectrometer (AB Sciex, United States). Ionization source parameters were set as follows: source temperature, 500°C; ion spray voltage, -4500 V; collision gas, 6 psi; curtain gas, 30 psi; and nebulizer gas and aux gas, 50 psi. Data were collected using a multiple reaction monitor (MRM).

BA data were analyzed using orthogonal partial least squares-discriminant analysis (OPLS-DA). Model performance was determined using the cross-validation parameters Q2 and R2Y. Metabolites with value importance in projection (VIP) > 1 were considered potential biomarkers for distinguishing different groups. BAs associated with AAD were selected using a random forest algorithm. The most important features were expressed as the mean decrease accuracy index exported through random forest analysis.

### Data analysis

Data were statistically analyzed using SPSS 21.0 and expressed as mean ± standard deviation (SD). Statistical analysis was performed using the Mann-Whitney-Wilcoxon and Kruskal-Wallis tests. Significance threshold was set as *P* < 0.05. Spearman’s correlation coefficient analysis was performed to clarify the correlation between the gut microbiota and BAs and visualized using a heatmap. Bar charts were generated using Origin 2022.

## Results

### QWBZP-TG-mediated regulation of gut microbiota diversity, structure, and abundance

**Figure 1:**
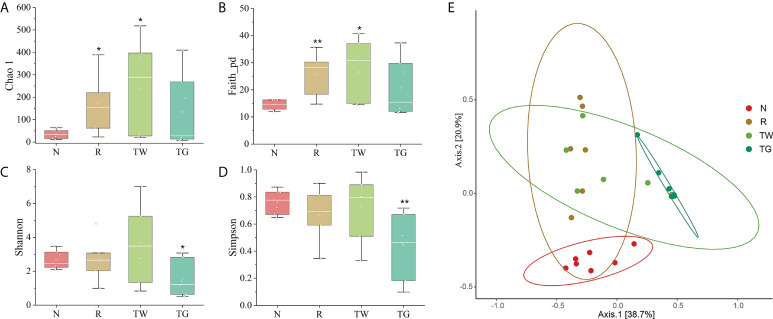
Effects of QWBZP-TG on the microbial diversity in AAD mice. **(A)** Chao 1, **(B)** Faith_pd, **(C)** Shannon, and **(D)** Simpson are the alpha diversity indices. **(E)** Principal coordinate analysis (PCoA) plot of the microbiota in the small intestine in the four groups (beta diversity). N, normal control group; R, antibiotic-associated diarrhea (AAD) restore group; TW, Qiwei Baizhu Powder (QWBZP) decoction group; TG, total glycosides of Qiwei Baizhu Powder (QWBZP-TG) group. Data are expressed as mean ± standard deviation (SD), *n* = 7. ^*^ *P* < 0.05 and ^**^ *P* < 0.01 compared with the N group.

The diversity and structural changes in the gut microbiota in response to QWBZP-TG administration were determined using 16S rRNA gene sequence. To comprehensively analyze the effects of QWBZP-TG on the microbial diversity of AAD mice, Chao1, Faith_pd, Shannon, and Simpson indices were calculated. Alpha diversity analysis revealed that administration of mixed antibiotics and QWBZP decoction resulted in increased Chao1 (*P* < 0.05 or *P* < 0.01), Faith_pd (*P* < 0.05), and Shannon (*P* > 0.05) (compared with normal mice; **Figures 1A–C**). However, QWBZP-TG administration resulted in remarkably reduced Shannon and Simpson (compared with normal mice; **Figures 1C**, **D**). The results indicated that antibiotics, QWBZP decoction, and QWBZP-TG disturbed intestinal microecology in mice. Intriguingly, the effects of QWBZP decoction and QWBZP-TG on the microbial diversity of AAD mice were inconsistent.

PCoA revealed that antibiotics, QWBZP decoction, and QWBZP-TG induced changes in the structure of the microbial community in mice (**Figure 1E**). The bacterial community profiles corresponding to the TG group were clustered away from those corresponding to the N and R groups. The microbes in the R and TW groups were more closely related. These results indicated that the intervention with mixed antibiotics resulted in altered gut microbiota structure in normal mice. Although treatment with both QWBZP decoction and QWBZP-TG resulted in altered microbial composition in mice with diarrhea, QWBZP-TG exhibited a more potent effect (compared with QWBZP decoction).

**Figure 2:**
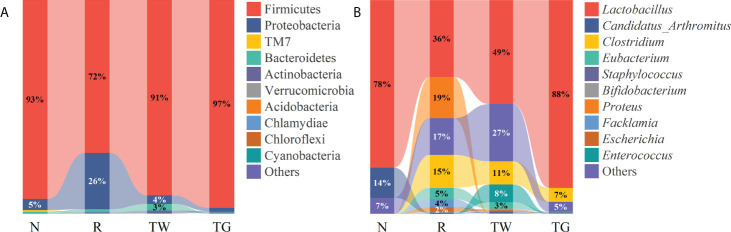
Effects of QWBZP-TG on the microbial structure in AAD mice. Relative mean abundance of small intestine microbiota at the **(A)** phylum and **(B)** genus levels in the four groups (*n* = 7). N, normal control group; R, antibiotic-associated diarrhea (AAD) restore group; TW, Qiwei Baizhu Powder (QWBZP) decoction group; TG, total glycosides of Qiwei Baizhu Powder (QWBZP-TG) group.

The relative abundance of gut microbiota was further evaluated at various taxonomic levels (**Figure 2**). At the phylum level, 18 phyla were detected in all samples. Among them, Firmicutes, Proteobacteria, TM7, Bacteroidetes, Actinobacteria, and Verrucomicrobia were shared among the four groups (**Figure 2A**). Firmicutes were the dominant bacteria accounting for the largest proportion. Compared with normal mice, the relative abundance of Firmicutes reduced sharply, while that of Proteobacteria increased markedly in AAD mice. The relative abundance of the two phyla returned to the baseline after treatment with the QWBZP decoction and QWBZP-TG. Moreover, the relative abundance of Bacteroidetes increased after treatment with mixed antibiotics and the QWBZP decoction.

**Figure 3:**
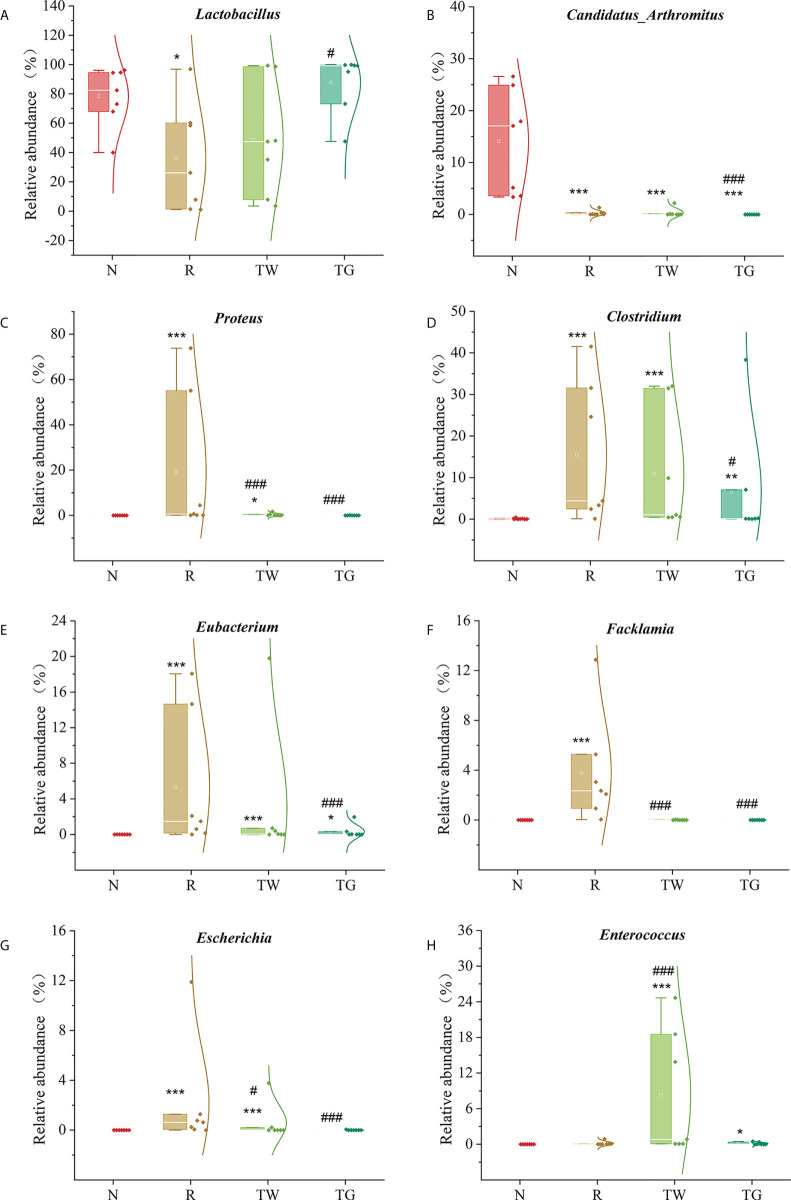
Relative abundance of signature genera. N, normal control group; R, antibiotic-associated diarrhea (AAD) restore group; TW, Qiwei Baizhu Powder (QWBZP) decoction group; TG, total glycosides of Qiwei Baizhu Powder (QWBZP-TG) group. Data are expressed as mean ± standard deviation (SD), *n* = 7. ^*^ *P* < 0.05, ^**^ *P* < 0.01, and ^***^ *P* < 0.001 compared with the N group; ^#^ *P* < 0.05 and ^###^ *P* < 0.001 compared with the R group.

**Figure 4:**
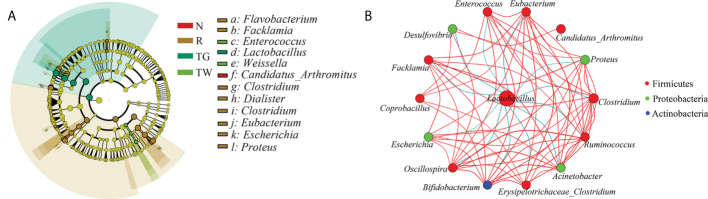
Core differential bacteria analysis of small intestine microbiota. **(A)** Cladogram of linear discriminant analysis effect size (LEfSe) analysis at the genus level (linear discriminant analysis [LDA] score > 4). N, normal control group; R, antibiotic-associated diarrhea (AAD) restore group; TW, Qiwei Baizhu Powder (QWBZP) decoction group; TG, total glycosides of Qiwei Baizhu Powder (QWBZP-TG) group. **(B)** Network of the correlation between major genera. The color of each spot corresponds to different phyla. Red and blue lines represent positive and negative correlation, respectively.

At the genus level, the abundance of *Lactobacillus* and *Candidatus*_*Arthromitus* (the major genera in the normal group) was reduced in AAD mice; conversely, the abundance of *Proteus*, *Clostridium*, *Eubacterium*, *Facklamia*, and *Escherichia* was increased in AAD mice (**Figure 2B**). The QWBZP decoction and QWBZP-TG could regulated the abundance of all these genera except *Candidatus*_*Arthromitus*, and QWBZP-TG was more remarkable than the QWBZP decoction at this regulation (**Figures 3A–G**). Moreover, differential bacteria at the genus level were identified through LEfSe analysis with linear discriminant analysis (LDA) score > 4 (**Figure 4A**). A total of 12 signature genera were identified and might be considered as the key bacteria in response to treatment with antibiotics, QWBZP decoction and QWBZP-TG. Among them, *Candidatus*_*Arthromitus* was the dominant bacteria in normal mice. *Clostridium*, *Eubacterium*, *Escherichia*, *Facklamia*, and *Proteus* (relative abundance > 2%) were the characteristic bacteria in AAD mice. The abundance of *Enterococcus* was higher in the mice treated with QWBZP decoction than in the other mice (**Figure 3H**), while *Lactobacillus* was a characteristic bacterium responding to QWBZP-TG (**Figure 4A**). The correlation between the top 15 genera (based on relative abundance) was calculated using Spearman’s correlation coefficient analysis (**Figure 4B**). *Lactobacillus* was negatively correlated with most genera (such as *Proteus*, *Clostridium*, *Eubacterium*, *Enterococcus*, *Escherichia*, *Desulfovibrio*, *Facklamia*, *Oscillospira*, *Bifidobacterium*, *Acinetobacter*, and *Ruminococcus*), indicating that it might be a key genus for maintaining intestinal microecology homeostasis.

### Regulation of BAs with QWBZP-TG

**Figure 5:**
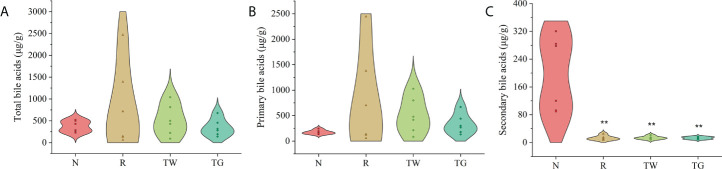
Effects of QWBZP-TG on the bile acid **(BA)** contents in AAD mice. **(A)** Total BAs, **(B)** Primary BAs, and **(C)** Secondary BAs. N, normal control group; R, antibiotic-associated diarrhea (AAD) restore group; TW, Qiwei Baizhu Powder (QWBZP) decoction group; TG, total glycosides of Qiwei Baizhu Powder (QWBZP-TG) group. ^**^ P < 0.01 compared with the N group.

LC-MS was used to analyze the BA content in colonic feces and to determine the effects of QWBZP-TG on the BA profile. The BA profile in AAD mice revealed high levels of total and primary BAs and low levels of secondary BAs (**Figure 5**). Moreover, higher levels of conjugated BAs and lower levels of unconjugated BAs were observed in AAD mice (compared with normal mice). Treatment with the QWBZP decoction and QWBZP-TG did restrict the abnormalities in the BA profile, but the values did not return to baseline (**Figure 5**). Surprisingly, no significant difference was observed between the normal and AAD groups in terms of the BA profile, indicating substantial within-group differences in AAD group.

**Figure 6:**
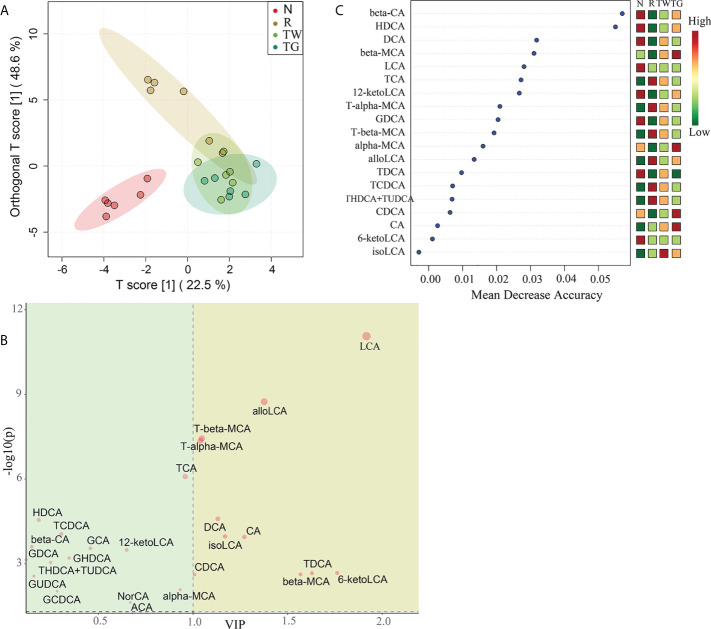
Differential bile acids (BAs) analysis of small intestine microbiota in the four groups. **(A)** Orthogonal partial least squares-discriminant analysis (OPLS-DA) scores plot. **(B)** OPLS-DA features. The dots in yellow area represent BAs with VIP >1 and *P* < 0.05. **(C)** Random forest analysis of BAs among the four groups based on mean decrease accuracy. The contents of each BA in different groups are represented by the shade of color. N, normal control group; R, antibiotic-associated diarrhea (AAD) restore group; TW, Qiwei Baizhu Powder (QWBZP) decoction group; TG, total glycosides of Qiwei Baizhu Powder (QWBZP-TG) group.

OPLS-DA was used to identify potential biomarker candidates associated with AAD and QWBZP-TG efficacy (R2Y=0.774, Q2 = 0.727). OPLS-DA revealed significant cluster separation within the normal and AAD groups, while the samples in the QWBZP decoction and QWBZP-TG groups did not exhibit marked cluster separation (**Figure 6A**). Our results showed that eleven BAs, including LCA, 6-ketolithocholic acid acetate (6-ketoLCA), tauroursodeoxycholic acid (TDCA), beta-MCA, allo lithocholic acid (alloLCA), CA, isolithocholic acid (isoLCA), DCA, tauro-alpha-muricholic acid (T-alpha-MCA), tauro-beta-muricholic acid (T-beta-MCA), and CDCA, with VIP >1 and *P* < 0.05, might serve as biomarkers in AAD and QWBZP-TG efficacy (**Figure 6B**). The BA contents were further evaluated using random forest analysis that revealed beta-CA, hyodeoxycholic acid (HDCA), and DCA to be the top three most important BAs (**Figure 6C**).

**Figure 7:**
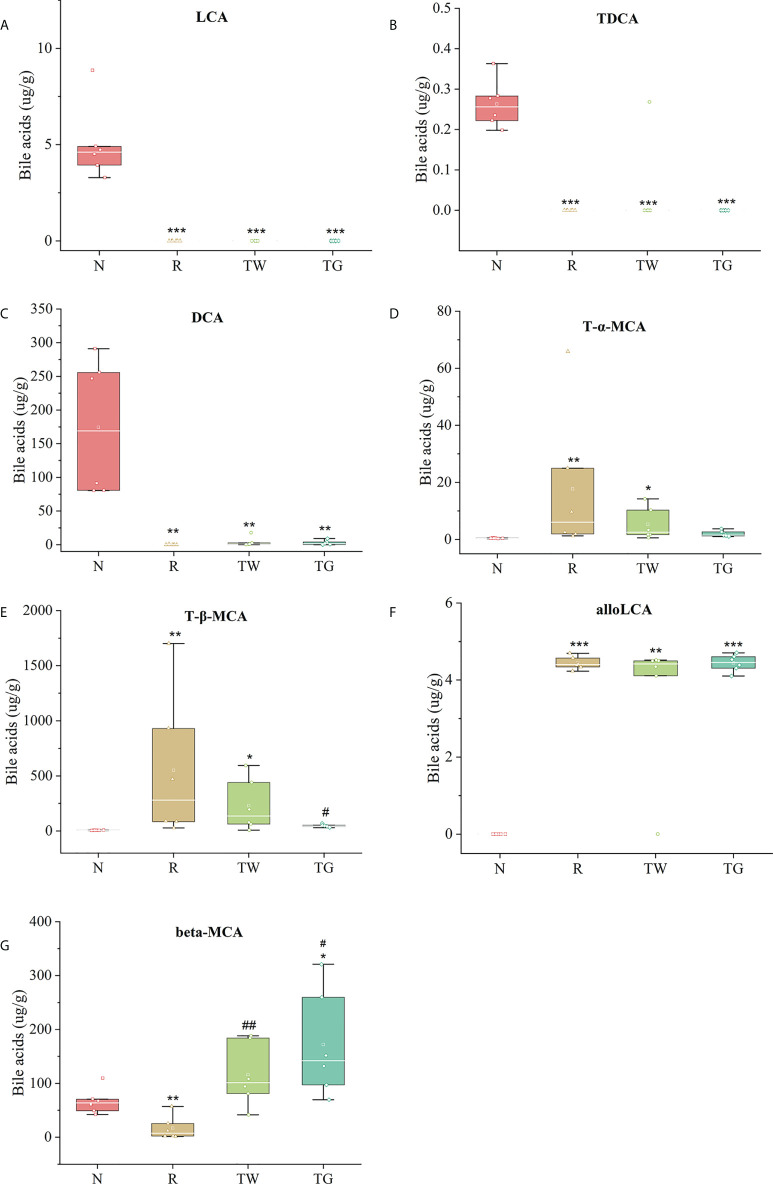
Contents of biomarker candidates of bile acid. ^*^ *P* < 0.05, ^**^ *P* < 0.01, and ^***^ *P* < 0.001 compared with the N group; ^#^ *P* < 0.05 and ^##^ *P* < 0.01 compared with the R group. N, normal control group; R, antibiotic-associated diarrhea (AAD) restore group; TW, Qiwei Baizhu Powder (QWBZP) decoction group; TG, total glycosides of Qiwei Baizhu Powder (QWBZP-TG) group.

LCA, DCA, alloLCA, TDCA, beta-MCA, T-alpha-MCA, and T-beta-MCA were shared between OPLS-DA and random forest analyses. Among them, LCA and TDCA were only detected in normal mice (**Figures 7A, B**). DCA, which is a secondary BA, was significantly more abundant in the N group than in the other groups (**Figure 7C**). Relatively high concentrations of T-alpha-MCA and T-beta-MCA (conjugated primary BAs) were detected in AAD mice, and they were reduced upon treatment with the QWBZP decoction and QWBZP-TG. Importantly, the levels of T-alpha-MCA and T-beta-MCA were lower in mice treated with QWBZP-TG than that treated with QWBZP decoction (**Figures 7D, E**). AlloLCA was detected in mice treated with mixed antibiotics, QWBZP decoction, and QWBZP-TG except in normal mice (**Figure 7F**). Furthermore, the concentrations of beta-MCA, an unconjugated primary BA in mice, were higher in TW and TG groups than in the N and R groups (**Figure 7G**).

### Correlation between gut microbiota and BAs

**Figure 8:**
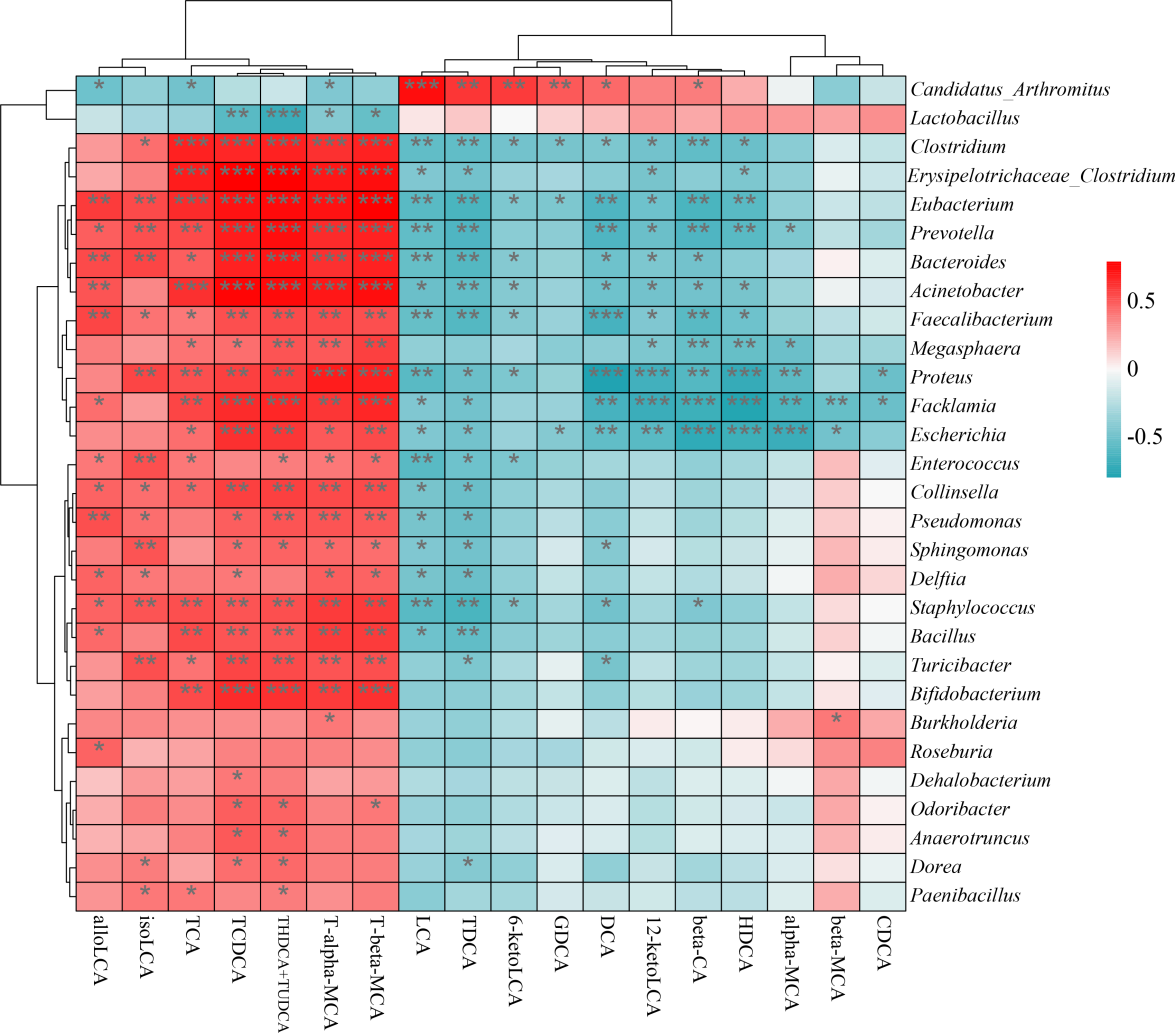
Correlation analysis investigating the association between gut microbiota and bile acids. Correlation coefficients are represented by the shades of color. Red and blue indicate positive and negative correlation, respectively. ^*^ *P* < 0.05, ^**^ *P* < 0.01, and ^***^ *P* < 0.001.

To evaluate the relationship between gut microbiota and BAs, we established a correlation heatmap using Spearman’s correlation coefficient analysis. As shown in **Figure 8**, most of the bacteria, especially *Eubacterium*, *Acinetobacter*, *Clostridium*, and *Proteus*, were positively correlated with T-alpha-MCA, T-beta-MCA, and TCDCA. However, *Escherichia*, *Facklamia*, and *Proteus* were negatively correlated with beta-CA, HDCA, and DCA, respectively. *Candidatus*_*Arthromitus* and *Lactobacillus* were positively correlated with most secondary BAs, such as LCA, DCA, 6-ketoLCA, 12-ketoLCA, GDCA, and TDCA, indicating their important role in BA metabolism.

## Discussion

AAD is one of the most frequent side effects associated with antibiotics used. Although the mechanism of AAD is complicated, the most common mechanism is antibiotic-induced microbial dysbiosis leading to altered metabolic function and decreased colonization resistance (Silverman et al., 2017). Thus, regulation of the gut microbiota and restoring intestinal homeostasis are central to diarrhea treatment (Li et al., 2021). Gut microbiota can be categorized into lumen- and mucosa-associated microbiota based on the different location occupied. Lumen-associated microbiota, which is a component of the intestinal fluid and does not come in direct contact with epithelial cells, is greatly affected by diet and drugs. It can directly participate in the metabolism of exogenous substances (Tropini et al., 2017). Current gut microbiome studies largely relied on colonic or feces samples. However, small intestine is the central organ for digestion, nutrient absorption, and immune function (Leite et al., 2020). In this study, QWBZP-TG-induced changes in lumen-associated microbiota of small intestine (at the level of diversity, structure, and abundance) were investigated.

Although reduced microbial diversity was commonly observed after antibiotics treatment, our result is inconsistent with previous studies. These discrepancies are likely due to differences in the samples used to assess the microbiome. Shannon and Simpson indices are used to reflect the species diversity. Intriguingly, the changes of Shannon and Simpson indices of AAD mice were controversial (compared with normal mice, **Figures 1C**, **D**), in part due to the difference in analysis method. Shannon index is more sensitive to the richness change than Simpson index, while Simpson index is more sensitive to the evenness change than Shannon index (Xu et al., 2011). Our finding indicated that AAD treatment may increase the richness rather than evenness of small intestine microbiota. Additionally, microbial diversity in mice treated with QWBZP decoction was relatively higher than that in AAD mice. However, the effect of QWBZP-TG on microbial diversity in AAD mice was contradictory to that of the QWBZP decoction (**Figures 1A–D**), possibly because QWBZP-TG regulated the growth of some specific bacteria, such as *Lactobacillus*. It is important to note that reduced diversity does not necessarily mean a reduced number of bacteria overall (Ramirez et al., 2020). Panda et al. (2014) reported that the total microbial load may increase after antibiotic treatment, even though microbial diversity is reduced. Therefore, marked changes in the abundance of specific taxa, rather than a loss of diversity, were associated with intestinal inflammatory diseases (Misic et al., 2018).

Alterations in microbial structure and abundance are responsible for the changes in metabolism and colonization resistance. *Lactobacillus*, a core bacterial genus in the small intestine of normal mice, markedly decreased after antibiotics treatment; meanwhile, diarrhea-associated bacteria such as *Clostridium* and *Escherichia* increased in AAD mice (**Figure 2**). *Lactobacillus* can antagonize pathogenic bacteria infection in the gut through bacteriocin, organic acids, and hydrogen dioxide (Huang et al., 2022). Thus, decreased *Lactobacillus* might diminish the resistance to pathogenic bacteria. Consequently, pathogens have the potential for overgrowth and intestinal domination during dysbiosis. In this study, correlation analysis also revealed that *Lactobacillus* exhibited negatively regulatory effects on most genera (**Figure 4B**). The abundance of *Lactobacillus* increased while the abundance of *Clostridium* and *Escherichia* reduced after treatment with QWBZP-TG (**Figures 3A**, **D**, **G**). These findings can support the aforementioned hypothesis.

BAs are considered endogenous laxatives. Primary BAs are absorbed in the small intestine. Bile salt hydrolases (BSH) mediate the gateway reaction of secondary bile acid production by hydrolyzing the C-24 N-acyl bond that binds bile acids to taurine or glycine; these reactions occur mostly in the lower small intestine and proximal colon (Cai et al., 2022). Poor BA absorption results in a large amount of efflux in the intestinal lumen, thereby inducing accelerated colonic movement and visceral hypersensitivity; this ultimately results in the occurrence of diarrhea (Duboc et al., 2012). Furthermore, abnormally high levels of BAs induce intracellular and extracellular cytotoxicity (Gärtner et al., 2015). Clinical studies have shown that patients with recurrent Crohn’s disease, diarrhea-type irritable bowel syndrome, or chronic idiopathic diarrhea exhibit BAs malabsorption and dysmetabolism, which are mostly characterized by increased levels of total BAs, relatively high levels of host-derived primary BAs, and low levels of microbially derived secondary BAs (Joyce and Gahan, 2017; Sinha et al., 2020). In this study, the levels of total and conjugated primary BAs in the colon of AAD mice were higher than those in normal mice (**Figure 5**). Consistent with the results of Zhang et al. (2014) and Theriot et al. (2014), our finding indicated that treatment with mixed antibiotics resulted in disturbed gut microbiota and disrupted BA metabolism, and caused BAs malabsorption in mice; consequently, large amounts of unabsorbed and unmetabolized BAs were passed on to the colon. However, the QWBZP decoction and QWBZP-TG reduced the total and conjugated primary BA levels. Particularly, the total BAs level returned to normal after treatment with QWBZP-TG (**Figure 5**), suggesting that excessive excretion of BAs was effectively controlled.

Impaired BA metabolism is associated with a decrease in Firmicutes abundance (Lavelle and Sokol, 2020). Bacteria within Firmicutes (especially *Lactobacillus*) play an important role in BA deconjugation as they harbor BSH genes (Enright et al., 2018; Winston and Theriot, 2020). Oral administration of some *Lactobacillus* strains, such as *L. reuteri*, *L. acidophilus*, *L. plantarum*, *L. paracasei*, and *L. bulgaricus*, results in increased levels of unconjugated BAs (Jones et al., 2012; Degirolamo et al., 2014). In addition, BAs are important regulators in the life history of *Clostridium difficile*. For example, the spore germination and outgrowth of *Clostridium difficile* are promoted by TCA (primary BA) but inhibited by DCA (secondary BA) (Silverman et al., 2017; Abbas and Zackular, 2020; Theriot and Petri, 2020; Wexler et al., 2021). These findings were also supported by the correlation analysis of gut microbiota and BAs in this study. Thus, the reduction in Firmicutes abundance in response to treatment with mixed antibiotics was responsible for the significant increase in the levels of primary BAs in the colon of AAD mice. In turn, this increase promoted the proliferation of *Clostridium difficile*. These results further suggested that QWBZP-TG could regulate the gut microbiota while gradually repairing disrupted BA metabolism. This regulatory effect might be one of the mechanisms underlying QWBZP-mediated diarrhea treatment.

TG is regarded as a key component in QWBZP promoting the growth of beneficial bacteria. The promoting effect of QWBZP-TG with respect to beneficial bacteria may be related to intestinal metabolism. Once glycosides enter the intestine, lumen-associated bacteria specifically respond to them and secrete glycosyl hydrolase to cleave the glycosidic bonds in glycosides, resulting in the release of saccharides and aglycones (Xu et al., 2017). Saccharides can then be used as energy sources to promote the growth of specific bacteria (Braune and Blaut, 2016). Several studies have highlighted the probiotic role of glycosides in the gut microbiota. Anthocyanins and their metabolites significantly promote the proliferation of *Bifidobacteria* and *Lactobacillus* (Hidalgo et al., 2012). *Akkermansia muciniphila* is enriched in high-fat diet-fed mice treated with puerarin (Wang et al., 2019). Flavone glycosides and triterpenoid saponins are dominant compounds in QWBZP-TG, and deglycosylation is the main type of glycoside hydrolysis that occurs in the intestine (Xie et al., 2022). Consequently, high levels of free glycosyls are produced to stimulate the proliferation of *Lactobacillus* and suppress the invasion of diarrhea-associated bacteria. Notably, QWBZP contains components other than glycosides that interact with glycosides to influence the effect of glycosides.

## Conclusion

QWBZP-TG exhibited physiological effects, including promotion of *Lactobacillus* proliferation, inhibition of infection with diarrhea-associated bacteria, and regulation of BA metabolism in AAD mice. These effects were consistent with those of QWBZP decoction; however, the effects were more significant in the case of QWBZP-TG. Thus, TGs are QWBZP components that contribute to the regulation of *Lactobacillus*, *Clostridium*, and *Escherichia* growth in the intestinal lumen and the TCA, DCA, beta-MCA, T-alpha-MCA, and T-beta-MCA contents in BAs.

## Data availability statement

The data presented in the study are deposited in the NCBI repository, accession number PRJNA846967.

## Ethics statement

The animal experiments were approved by the Animal Care and Use Committee of Hunan University of Chinese Medicine (authorization number: LL2020102103).

## Author contributions

GX conceived, designed and performed the experiments, and wrote the manuscript; ND performed the experiments; TZ and XP analyzed the data; SZ and ZT supervised the work and reviewed the manuscript. All authors contributed to the article and approved the submitted version.

## Funding

This work was supported by the National Natural Science Foundation of China (81804076), the Natural Science Foundation of Hunan Province (2020JJ5426), and First-class Discipline Project on Chinese Pharmacology of Hunan University of Chinese Medicine (No. 201803).

## Conflict of interest

The authors declare that the research was conducted in the absence of any commercial or financial relationships that could be construed as a potential conflict of interest.

## Publisher’s note

All claims expressed in this article are solely those of the authors and do not necessarily represent those of their affiliated organizations, or those of the publisher, the editors and the reviewers. Any product that may be evaluated in this article, or claim that may be made by its manufacturer, is not guaranteed or endorsed by the publisher.
